# Double-Wall Carbon Nanotube Hybrid Mode-Locker in Tm-doped Fibre Laser: A Novel Mechanism for Robust Bound-State Solitons Generation

**DOI:** 10.1038/srep44314

**Published:** 2017-03-13

**Authors:** Maria Chernysheva, Anastasia Bednyakova, Mohammed Al Araimi, Richard C. T. Howe, Guohua Hu, Tawfique Hasan, Alessio Gambetta, Gianluca Galzerano, Mark Rümmeli, Aleksey Rozhin

**Affiliations:** 1 Nanoscience Research Group and Aston Institute of Photonics Technologies Aston University, Aston triangle Birmingham, B4 7ET, UK; 2 Novosibirsk State University, Novosibirsk, 630090, Russia; 3 Institute of Computational Technologies SB RAS, Novosibirsk, 630090, Russia; 4 Al Musanna College of Technology, Muladdah, Al Musanna, Sultanate of Oman; 5 Cambridge Graphene Centre, University of Cambridge, Cambridge, CB3 0FA, UK; 6 Istituto di Fotonica e Nanotecnologie-CNR and Dipartimento di Fisica-Politecnico di Milano, Milano 20133, Italy; 7 Soochow Institute for Energy and Materials InnovationS, College of Physics, Optoelectronics and Energy & Collaborative Innovation Center of Suzhou Nano Science and Technology, Soochow University, Suzhou 215006, China; 8 Key Laboratory of Advanced Carbon Materials and Wearable Energy Technologies of Jiangsu Province, Soochow University, Suzhou, 215006, China; 9 IFW Dresden, P.O. Box 270116, 01069 Dresden, Germany; 10 Centre of Polymer and Carbon Materials, Polish Academy of Sciences, M. Curie-Sklodowskiej 34, Zabrze 41-819, Poland

## Abstract

The complex nonlinear dynamics of mode-locked fibre lasers, including a broad variety of dissipative structures and self-organization effects, have drawn significant research interest. Around the 2 *μ*m band, conventional saturable absorbers (SAs) possess small modulation depth and slow relaxation time and, therefore, are incapable of ensuring complex inter-pulse dynamics and bound-state soliton generation. We present observation of multi-soliton complex generation in mode-locked thulium (Tm)-doped fibre laser, using double-wall carbon nanotubes (DWNT-SA) and nonlinear polarisation evolution (NPE). The rigid structure of DWNTs ensures high modulation depth (64%), fast relaxation (1.25 ps) and high thermal damage threshold. This enables formation of 560-fs soliton pulses; two-soliton bound-state with 560 fs pulse duration and 1.37 ps separation; and singlet+doublet soliton structures with 1.8 ps duration and 6 ps separation. Numerical simulations based on the vectorial nonlinear Schr¨odinger equation demonstrate a transition from single-pulse to two-soliton bound-states generation. The results imply that DWNTs are an excellent SA for the formation of steady single- and multi-soliton structures around 2 *μ*m region, which could not be supported by single-wall carbon nanotubes (SWNTs). The combination of the potential bandwidth resource around 2 *μ*m with the soliton molecule concept for encoding two bits of data per clock period opens exciting opportunities for data-carrying capacity enhancement.

Ultrafast fibre lasers, as complex nonlinear systems, hold a variety of physical challenges and potential for engineering applications, influencing the development of metrology, micromachining, optical signal processing and fibre communications. The last of these is of particular research interest due to data “capacity crunch”. The emergence of low-loss (4.5 dB/km) hollow-core optical fibres^1^ has accelerated the development of 2 *μ*m fibre optic telecommunications^1^,^2^. Fibre optics, free-space and atmosphere communications, light detection and ranging (LIDAR) and remote sensing technologies have put a high demand on high-efficient fibre lasers and amplifiers around 2 *μ*m. This particularly applies to Tm-doped fibre sources.

Apart from the consideration on new carrier wavelengths, one of potential approaches to addressing the capacity problem is a coding alphabet extension. The coding concept of bound-state solitons suggests a data stream using four symbols: logical zero, one (single soliton), two (two-soliton molecule), and three (three-soliton molecule). Such a quaternary coding scheme (M = 4) doubles the data-carrying capacity of existing binary systems (M = 2), according to log_2_M law. Moreover, the bound-state soliton coding approach is compatible with all present techniques of multiplexing and modulation, allowing further capacity increase of all-optical bit storage^3^, all-optical buffers^4^ and telecommunications lines^5^,^6^.

Soliton molecule generation, alongside soliton bunching^7^ or harmonic mode locking^8^, occurs due to over-driving of mode-locked ultrafast fibre lasers. For example, during high-energy operation, the slope of the saturable absorber transmission changes to negative, causing higher losses for higher intensities^9^. The characteristic features of soliton molecules are: (i) a fixed temporal separation, bounding together two or more soliton pulses into a single pulse; (ii) small temporal separation of bound pulses, compared to the cavity round-trip time; (iii) a fixed phase difference of 0, ±*π* 10, or ±*π*/2 11,12. The formation of stable bound soliton states can be theoretically explained by two major mechanisms of soliton energy quantisation and balanced attraction of each soliton pulse. The highest energy of a fundamental soliton with duration *τ*_*p*_ is limited by the soliton area theorem: *E*_*s*_ ∝ |*β*_2_|/(*γ* · *τ*_*p*_), where *γ* is the net nonlinear coefficient and *β* is the total cavity dispersion. Soliton energy quantisation results in pulse splitting at operation powers higher than the fundamental limit^13^,^14^,^15^,^16^. The stable bound soliton pair is formed due to the interplay among phase modulation, anomalous group velocity dispersion (GVD), and the repulsive forces of adjacent split solitons during propagation along the laser cavity. As a result, the leading soliton will experience a negative frequency shift, accompanied by a decrease in its group velocity^17^. On the other hand, the trailing soliton will experience an increase in the group velocity. Such a soliton pair uses the dissipative nonlinear dynamics of the active cavity to remain stable for hours without the necessity for active stabilisation^18^,^19^. The practical implementation of the soliton molecules concept requires intensive investigation on the multi-soliton complexes, their propagation dynamics, formation or dissociation, mutual interactions and stability.

The other phenomenon arising from over-driving the mode-locking mechanism is a multi-wavelength generation^20^. A Multi-wavelength ultrafast generation is widely applicable to wavelength division multiplexing and optical signal processing in telecommunication, optical sensing, precision spectroscopy, and biomedical research. For a multi-wavelength generation, the cavity should possess an intensity or wavelength-dependent loss element. This relieves the mode competition produced by homogeneous gain broadening, for example, through the nonlinear optical Kerr effect (NPE or nonlinear optical loop mirrors)^21^. However, the main obstacle to multi-wavelength generation in Tm-doped fibre lasers is the strong homogeneous line broadening of active fibres (∼300 nm). Recently, *Z. Yan* *et al*. have presented a switchable and tuneable single-longitudinal-mode multi-wavelength Tm-doped fibre laser, based on NPE^22^,^23^, resulting in limited progress in the field.

Recently, numerous works have investigated stable soliton molecule formation and demonstrated their generation in various laser configurations, dispersion-managed and dissipative regimes, in agreement with numerical simulations^24^,^25^,^26^,^27^,^28^,^29^. Despite the significant theoretical and applied interest, the existence, characteristics, and dynamics of soliton molecules have not yet been extensively studied at a wavelength beyond 1.5 *μ*m^30^,^31^. Growing research interest in this topic^32^,^33^ underscores its high potential for application in telecommunication, metrology, trapping and manipulation of atoms and nanoparticles and control of magnetisation. However, achieving high flexibility in generation and control of the bound-state dynamics remain challenging to meet the requirements of these applications. We previously studied a Tm-doped ring fibre laser system mode-locked with SWNTs and NPE^34^. The laser generated 600 fs pulses at 72.5 MHz repetition rate with an average output power of 300 mW. Although the laser generates higher-order solitons at the highest available pump power, no stable multi-soliton generation has been observed. The major complication to obtaining tightly bound-state solitons with fixed separation and phase difference is small modulation depth and relatively slow relaxation time of typical material-based SAs used in the wavelength region around ∼2 *μ*m. Thus, they are not capable of suppressing random fluctuation of the relative phase, and therefore, random interaction between multiple solitons. The modulators based on optical nonlinear Kerr-effect have fast relaxation time. However, the low nonlinearity of silica fibres close to ∼2 *μ*m limits their performance. This motivates us to investigate other saturable absorbers with suitable parameters for stable multi-soliton formation in the 2 *μ*m wavelength region.

In this paper, we demonstrate both theoretical and experimental observation of stable bound-state solitons in Tm-doped fibre laser hybrid mode-locked employing NPE and DWNT-SA. We provide a comprehensive comparison between the conventional SWNTs and DWNTs as SA to initiate and support high-power conventional solitons and various types of stable soliton molecule generation close to the 2 *μ*m region or above. To characterise the multi-soliton complexes theoretically, we perform a numerical modelling based on a vectorial nonlinear Schrödinger equation (NLSE). The results validate the high potential of hybrid mode-locked Tm-doped fibre lasers employing DWNTs and underscore their superior performance as compared to SWNT mode-locked fibre lasers. We believe our demonstration presents new opportunities for data capacity enhancement and will show implication in scientific and biomedical areas.

## Materials and Methods

### Double-wall carbon nanotube saturable absorber

In the wavelength region from 1.4 to 3 *μ*m, material-based SAs commonly used to initiate ultrashort pulse generation are semiconductor saturable absorber mirrors (SESAM)^35^,^36^, SWNTs^37^,^38^,^39^,^40^,^41^,^42^, graphene^43^,^44^,^45^ and semiconducting transition metal dichalcogenides (s-TMDs) such as MoS_2_^46^, WS_2_^47^, MoSe_2_^48^, *etc*. In addition, ultrashort pulse generation can be ensured using mechanisms based on the nonlinear optical Kerr-effect, such as NPE^49^, nonlinear optical or amplifying loop mirrors (NOLM or NALM)^50^. Recently, a combination of material SAs and nonlinear effects has been used to demonstrate its potential for higher-power and higher-quality ultrashort pulse formation^34^,^51^,^52^. Though both graphene and s-TMDs offer wideband operation, they typically feature small modulation depths^45^,^53^. Beyond 1.3 *μ*m, SESAM performance is also limited by the growth of highly strained layers on GaAs-based Bragg mirrors due to the requirement to increase indium concentration^54^, decreasing the quality of the surface and increasing insertion losses.

DWNTs, formed by two concentric SWNTs^55^, offer strong third-order optical nonlinearity comparable to those of SWNTs^56^ and broadband absorption^57^. The combination of metallic (m-) and semiconducting (s-) inner and outer walls increases their carrier relaxation speed due to tunnelling from s- to m-tubes^58^. Of particular relevance to this work, DWNTs also exhibit low saturation intensity similar to that of the SWNTs^57^,^59^. DWNTs have higher thermal stability than SWNTs^55^, which may have significant implications when high power, high energy pulsed operation is envisaged. Recently, DWNT-SA have been integrated in fibre laser cavities and demonstrated attractive wide-band ultrafast pulse generation in the 1, 1.55 and 2 *μ*m regions^60^.

In our work, DWNTs are produced by catalytic chemical vapour deposition (CCVD)^61^ of CH_4_ over Mg_1−*x*_Co_*x*_O solid solution containing Mo oxide^62^. After growth, the nanotubes are oxidised in air at 570 °C for 30 min^63^. The residual material is next rinsed with hydrochloric acid (HCl) to dissolve the metal oxides^63^. We use a solution processing strategy to fabricate the free-standing DWNT-SAs, similar to that described in Ref. 57. Briefly, purified DWNTs (0.04 wt.%) are ultrasonicated in water with 1 wt% sodium dodecylbenzene sulfonate (SDBS) surfactant at 10 °C for 4 hours. The stable dispersion is next centrifuged at 100,000 *g* to remove the large bundles. 4 ml of the top 60% of the dispersion is then mixed with 1 wt% of aqueous polyvinyl alcohol (PVA) polymer solution. Slow evaporation of water at room temperature yields a ∼50 *μ*m free-standing DWNT-SA polymer composite.

### Optical transmission

**Figure 1:**
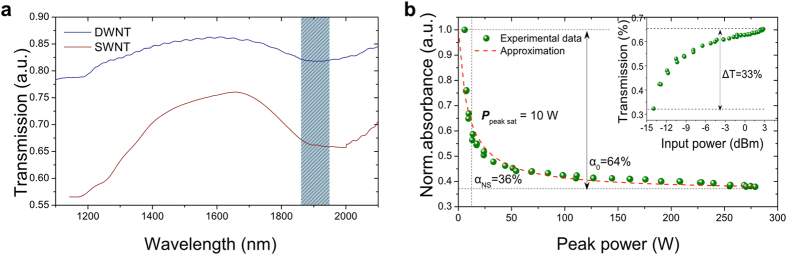
(**a**) IR transmission spectrum of DWNT-PVA composite film (blue line). The colour bar indicates the laser operation band, *i.e.*: 1860–1960 nm. (**b**) Power-dependent DWNTs absorption under the excitation of 600 fs pulses at 1.94 *μ*m: the circles are the experimental data and the solid curves are the analytical fit to the data.

Figure 1(a) shows the optical transmission spectrum of the DWNT-PVA composite with subtracted PVA background. PerkinElmer Lambda 950 spectrophotometer with 1 nm step is used to measure the IR optical transmission. From Fig. 1(a), one can distinguish two absorption bands (max at ∼1.1 *μ*m with ∼250-nm width and at ∼1.9 *μ*m with ∼350-nm width) for DWNT spectrum, which are a combination of excitonic transitions from both inner and outer walls of tubes^57^,^64^. This corresponds to tube diameter range from 0.75 to 1.15 nm (*eh*_11_ at ∼1.1 *μ*m) and 1.5 to 1.9 nm (*eh*_11_ at ∼2 *μ*m and *eh*_22_ at ∼1.1 *μ*m)^57^,^65^, respectively. This matches statistical DWNTs inner and outer diameter distributions. The 8% SWNTs present in the sample have a diameter range similar to that of the inner wall of DWNTs. Thus, *eh*_11_ absorption of these SWNTs also contributes to the ∼1.1 *μ*m absorption peak from the *eh*_11_ of inner and *eh*_22_ of the outer walls of DWNTs. However, no significant *eh*_11_ or *eh*_22_ absorption peaks from the inner walls of the triple wall carbon nanotubes are observed^57^. For comparison, the red plot in Fig. 1(a) presents optical transmission spectrum of the laser ablation SWNT-PVA, used in Ref. 34.

### Power-dependent measurements

We investigate the absorption saturation behaviour of the DWNT polymer composite SA. A home-made Tm-doped ultrafast fibre laser generating transform-limited 600 fs soliton pulses at 74 MHz repetition rate is used as the probe laser. The average laser power is controlled by a variable optical attenuator (VOA). Therefore the ultrafast laser output parameters are strictly fixed during the measurements. The laser output radiation after VOA is split into two equal parts *via* a 3-dB coupler, one of which acts as a reference arm and is measured directly *via* powermeter. Application of the VOA allows the average power launched at the arm containing the DWNT polymer film to be varied from several microwatts up to 3.5 mW, corresponding to a maximum pulse peak power of 380 W. Figure 1(b) shows power-dependent measurement of the DWNT-SA. As seen from Fig. 1(b), the normalised absorption *α* decreases with the increasing probe peak intensity according to the two energy level model, described by the following equation^66^:


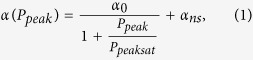


where *α*_0_ is the modulation depth, *α*_*ns*_ is non-saturable losses, *P*_*peak*_ is the launched peak power and *P*_*peaksat*_ is the saturation peak power *i.e.* the peak power necessary to reduce the absorption coefficient to half the initial value.

The obtained values of *α*_*ns*_, *α*_0_ and *P*_*peaksat*_ are 36%, 64% and 10 W, correspondingly (Fig. 1b). The corresponding increase of the sample linear transmission is ∼33%. The modulation depth *α*_0_ and the saturation peak power *P*_*peaksat*_ are significantly higher compared to laser ablation SWNT PVA-based thin film composites operating around ∼2 *μ*m (40% and 1.44 W, respectively)^34^.

### Pump-probe

The carrier dynamics of SWNT-SA have been studied over the past years, employing time-resolved spectroscopy, also known as the pump-probe technique^67^. However, the relaxation time of DWNTs has received limited attention thus far.

**Figure 2:**
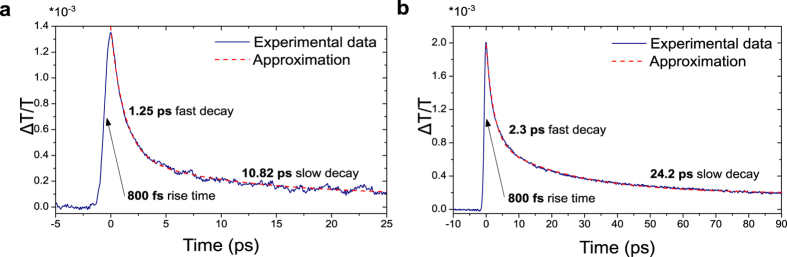
Pump-probe traces for (**a**) DWNTs, (**b**) SWNTs.

For the pump-probe measurements, we adopted an Asynchronous Optical Sampling (ASOPS) configuration^68^ based on a pair of mode-locked erbium-doped fibre lasers (Menlo System) operating at a repetition frequency *f*_*r*_ of 250 MHz, each equipped with a highly non-linear fibre for supercontinuum (SC) generation in the 1–2.3 *μ*m wavelength range. The long-wavelength portion of each SC output is tuned and filtered, matching the operation wavelength of Tm-doped fibre lasers, and to exploit only the first order soliton centred at ∼1880 nm. Each soliton pulse has a ∼65 fs duration and carries ∼20 mW average power (corresponding to 2 nJ pulse energy) at the output of the SC fibre, right before entering a 10 cm standard single mode fibre, equipped with a FC/PC connector. Both soliton output beams are focussed onto the sample: the first pulse-train acts as a pump exciting the sample while the second (attenuated to sub-mW power levels) probes the pump-induced population changes as a function of the pump-probe delay. When the repetition frequencies of the two lasers are slightly detuned, the two output pulses are automatically subjected to a temporal delay *τ* corresponding to an integer multiple of the repetition period difference Δ*τ*. The pump-probe delay therefore periodically increases from zero to a maximum value of 1/*f*_*r*_ (4 ns for a 250 MHz system) with a repetition period *T*_*scan*_. This corresponds to the inverse of the repetition frequency detuning (*T*_*scan*_ = Δ*f*_*r*_ − 1) which also sets the time-window for a single acquisition. For the present measurements, we adopt a frequency detuning Δ*f*_*r*_ ∼ 2.5 kHz between pump and probe pulses, corresponding to Δ*τ* ∼ 40 fs and *T*_*scan*_ ∼ 400 *μ*s. The pump-probe trace is recorded through a 500 MHz, 12-bit digital oscilloscope (Teledyne Lecroy), by shining the probe beam onto an extended InGasAs detector (60 MHz bandwidth, in a balanced configuration). Both pump and probe were focused with a spot size of 50 *μ*m onto the sample. Since the time delay is generated by the difference in the pulse repetition rates of pump and probe without any mechanical moving part, no artefacts are induced from misalignments during the scan such as beam-walk effects^68^. We also rule out both pump-induced nonlinear effects (such as two-photon absorption) and thermal-induced artefacts, thanks to the low energy-per-pulse and the single-trace very short acquisition time, respectively. Figure 2(a) demonstrates the temporal evolution of the population integrated over selected wavelength range.

We then compare the time-resolved spectroscopy results of the DWNTs with laser ablation SWNTs^69^, which were studied in our previous work^34^. The temporal dynamics, as shown in Fig. 2(b) indicates a 800 fs rise-time (due to a combination of the pulse duration, detector bandwidth and pulse-to-pulse time jitter) for the trace of both DWNT and SWNT samples. The temporal relaxation dynamics is a combination of a fast (1.25 and 2.3 ps), and a slow decay (10.82 and 24.2 ps), for DWNT and SWNT samples respectively. Therefore, the DWNT/PVA sample shows 2 times faster relaxation times than SWNT-SA. This underscores the promise of DWNTs as a fast saturable absorber in high-intensity laser optical cavities, operating close to the 2 *μ*m wavelength region. In particular, this could enable the generation of single pulses and stable multi-soliton complexes in the mode-locking regime.

### Design of the experimental setup

**Figure 3:**
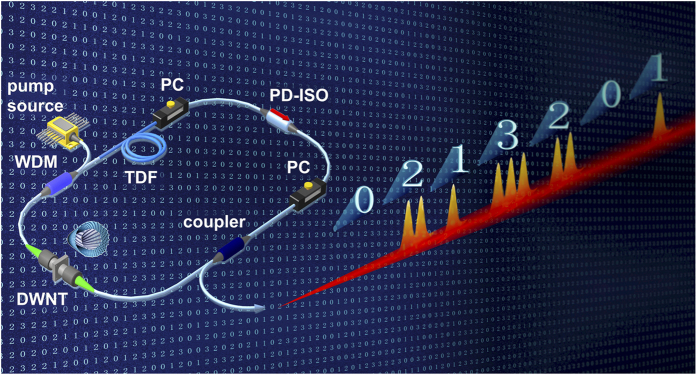
Schematic setup of the ring hybrid mode-locked Tm-doped fibre laser, demonstrating an example of quaternary coding using multi-soliton complexes. The cavity consists of: high concentration Tm-doped fibre (TDF), polarisation controllers (PCs), polarisation dependent isolator (PD-ISO), 3-dB output coupler, DWNT dispersed in PVA-based film sandwiched between two optical connectors, 1550/2000 isolating wavelength division multiplexer (WDM), and 1550 nm Fabry-Perot laser diode amplified by EDFA (pump source).

The as-prepared DWNT-SA is next incorporated into a passive mode-locked Tm-doped fibre laser to generate laser pulses. The laser setup follows the previously demonstrated concept of a hybrid mode-locked fibre laser^34^ and is presented in Fig. 3. The unidirectional ring laser cavity is ∼3 m long, comprising of a 1 m highly doped Tm-doped aluminum-silicate (0.8 wt% thulium, 3.6 wt% aluminum) fibre (from FORC RAS). The active fibre has a peak absorption of 60 dB m^−1^ and geometrical parameters similar to the standard SMF-28 fibre: a 10 *μ*m core and 125 *μ*m cladding diameters. The Tm-doped fibre has a nominal group velocity dispersion (GVD) of *β*_2_ = −76 ps^2^ km^−1^ at 1.9 *μ*m. The active fibre features a cut-off wavelength *λ*_*c*_ of 2.2 *μ*m, nominal absorption at the central operation wavelength 1885 nm of 20.5 dB km^−1^ and Kerr nonlinear coefficient 0.78 W^−1^ km^−1^.

The 1550 nm Fabry-Perot continuous-wave laser diode, amplified by erbium-doped fibre amplifier (EDFA) from IPG Photonics to up to 1.2 W maximum power, provides pump light through the high isolation wavelength division multiplexer (WDM). The output of the laser is extracted *via* a 40% fibre coupler for further pulse characterization. The ports of the passive components, (*i.e.* isolator, coupler and WDM) were pigtailed with conventional SMF-28 fibre, with a combined length of 1.7 m. The SMF-28 fibre GVD, losses and nonlinearity are −74 ps^2^ km^−1^, 14.11 dB km^−1^ and 0.78 W^−1^ km^−1^ in the 1.88 *μ*m wavelength band, respectively. Because the Tm-doped fibre and SMF-28 fibres have similar mode field diameter, the splicing losses are negligible (less than 0.02 dB).

Both the NPE and DWNT-SAs are used to achieve self-started mode locking operation in the laser. The NPE is formed by a polarisation-dependent isolator (PD-ISO), triggered by adjusting the pair of the polarisation controllers (PC). Due to low photon energy close to 2 *μ*m wavelength range (compared to 1–1.5 *μ*m bands), the threshold of nonlinear optical Kerr-effect is higher. Therefore mode-locking initiation *via* NPE in Tm-doped fibre lasers is also difficult. As a result, with the current laser cavity configuration (*i.e.* cavity length of 3 m) and available pump power (1.2 W), mode-locking cannot self-start using NPE only. A DWNT-SA can independently initiate mode-locking. However NPE, as additional pulse formation mechanism, ensures the overall laser generation stabilisation. Therefore, application of a hybrid mode-locking configuration enables ultrashort pulse generation with significantly higher average powers, higher temporal purity and frequency stability^70^. This also reduces the possibility of thermal degradation of the DWNT-SA polymer nanocomposite sandwiched between fibre ferrules^34^. Indeed, alternative methods to enhance carbon nanotubes thermal damage threshold have been demonstrated based on evanescent field interaction, for example, by using fibre tapers^71^, D-shaped^72^ or hollow core fibres^73^. Though these methods also allow a distributed SA with increased interaction length, fabrication of such devices is more complicated and is time and resource-consuming.

### Measurement facilities for laser performance characterisation

The optical spectrum analyser we use in the experiment is from Yokogawa optical spectrum analyser (OSA) with a range of 1700–2400 nm and 0.05 nm resolution. The photodiode used to measure the laser pulse is from Photonics Solutions (ET-5000F) with a bandwidth of 12.5 GHz. The autocorrelator is from Avesta (AA-20DDR), and oscilloscope is from Le Croy (WJ352A) with the sample rate of up to 2 GS s^−1^. The output power is characterised using a photodiode from Thorlabs (PM100D with S302C).

### Numerical Model

For numerical modelling of the hybrid mode-locked laser, shown in Fig. 3, we take into account the discreteness of its intracavity elements. The pulse propagates through different elements consequently. The fibre laser scheme used in the simulation consists of an active Tm-doped fibre, a passive SMF-28 fibre, an output coupler, a SA, a PD-ISO and a PC according to experimental setup (Fig. 3). The effects of mode-locking and filtering in polarisation dependent isolator are treated as point-action, while the evolution of electromagnetic field amplitude *E(z,t)* inside the passive fibre is described with vectorial NLSE^14^,^74^:


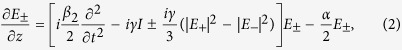


where 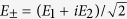 are right-hand and left-hand circular polarisation of the electric field, *I* = |*E*_+_|^2^ + |*E*_−_|^2^ is signal intensity, *α* = 14 dB km^−1^ is the optical losses, *β*_2_ = −74 ps^2^ km^−1^ is the second-order dispersion coefficient at the central frequency *ω*_0_, *γ* = *n*_2_*ω*_0_/(*cA*_*eff*_) = 0.78 W^−1^ km^−1^ is the Kerr nonlinearity coefficient with the nonlinear refractive index *n*_2_ and the effective fibre cross-section area *A*_*eff*_ for a fundamental mode. The equation is solved by using the split-step Fourier-transform method^13^. The simulations are run until the optical field reaches the steady state after a certain number of cavity round trips, taking into consideration the contribution of the point-action elements described below.

The numerical model for amplification in the 1-m long Tm-doped active fibre uses a similar NLSE model with additional accounting for the effects of gain filtering and saturation. The gain saturation depends on the total pulse energy:


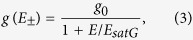


where *E* is the signal energy given by 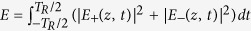 and 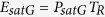 is the saturation energy, 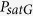 is saturation power of the gain, *T*_*R*_ is the cavity round trip time, *g*_0_ is the small signal gain coefficient of 40 dB m^−1^ with total gain 40 dB. The wavelength dependence of the gain is implemented in the frequency domain using the Lorentzian line shape with a bandwidth of 100 nm and a central wavelength of 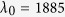 nm. The amplifier noise is simulated by an additive complex “white” noise. The values of the nonlinear parameter dispersion and losses of the Tm-doped fibre used in the numerical modelling are *β*_2_ = −76 ps^2^ km^−1^, *γ* = 0.78 W^−1^ km^−1^, *α* = 2.54 dB m^−1^.

The response of saturable absorber on instantaneous pulse power within each round-trip is given by Ref. 75:


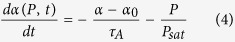


where *P* = |*E*_±_|^2^ - is the incident power of the input optical pulse, and *τ*_*A*_ - is the relaxation time of a SA. In case of fast absorber, the saturable absorption recovers immediately and equation is reduced to Eq. (1). The linear limits of saturable absorption and non-saturable absorption of DWNT-SA are *α*_0_ = 0.64 and *α*_*ns*_ = 0.36, respectively, while the saturation power *P*_*sat*_ = 10 *W*.

To describe signal propagation through the polarisation-dependent isolator the circular-polarisation should be rewritten via linear-polarisation. After passage through polarisation-dependent isolator one linearly-polarised component goes out of the cavity *via* output port and no longer propagates in the cavity. On the other hand, the orthogonal polarisation component modified by polarisation controller enters the fibre part of the cavity. The simplified model of polarisation controller includes half (HWP) and quarter wave plates (QWP) with varying operating angles *χ* and *ψ*, which enables control of light ellipticity and angle of the polarisation ellipse axis:


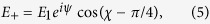



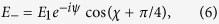


Note that we do not take into account the action of polarisation controller located before polarisation-dependent isolator to simplify the numerical model and reduce the number of parameters.

## Results and Discussion

### Simulation results

**Figure 4:**
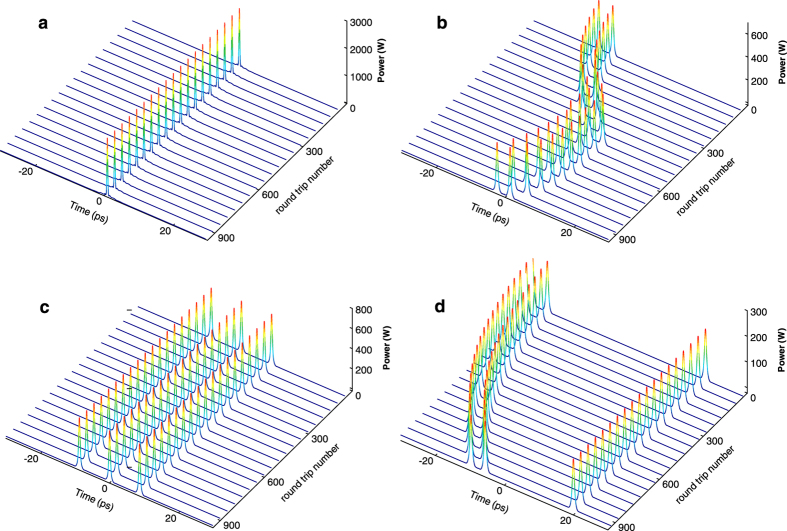
Spatio-temporal dynamics of (**a**) single- and (**b–d**) multi-soliton regimes of laser generation in numerical simulation. The spatio-temporal dynamics is calculated at the laser output over 1000 round trips in steady-state.

**Figure 5:**
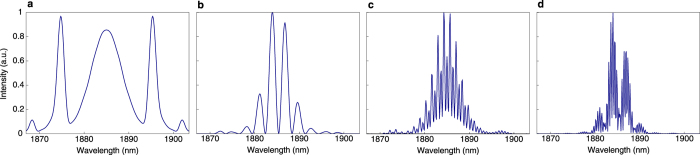
Output spectra of (**a**) single- and (**b–d**) multi-soliton regimes of laser generation in numerical simulation corresponding to spatio-temporal dynamics shown in Fig. 4.

Figures 4 and 5 show numerical simulation of different regimes of DWNT mode-locked laser generation. Shorter relaxation time of DWNT-SA (1.23 ps) facilitates phase-locking and, therefore, allows the transition from single-pulse soliton (Figs 4a and 5a) to two-soliton bound states generation (Figs 4b and 5b) with the variation in operating angles of the HWP and QWP at fixed pump power. Distance and phase difference between the output pulses depend on the polarisation state adjustment and could fluctuate depending on the round trip number. Further increase in pump power leads to the generation of multi-soliton solutions. A three-soliton solution with pulses moving with the same constant velocity along the *t* axis is shown in Figs 4c and 5(c). Figure 4(d) shows more complicated dynamics when soliton pair and a single soliton exist simultaneously in the cavity. In this instance, a stable pair has group velocity different from the velocity of a single soliton. We note that group interactions of such solitons were thoroughly studied in Ref. 26. The experimental study of the single- and multi-pulse regimes of generation are in close agreement with results of the numerical simulations.

Analogous simulation is implemented for SWNT mode-locked Tm-doped fibre laser. The simulations for both SWNT and DWNT mode-locked laser schemes are performed under identical conditions except for parameters of saturable absorbers. Multi-soliton generation is observed in both cases while formation of tightly bound-state solitons (with stable temporal separation of bound pulses which is less than five pulse widths^10^,^19^,^25^,^30^) is observed only with the parameters of DWNT-SA.

The simulation results proved superior properties of DWNT-SAs, when compared with SWNT ones. The DWNT-SA plays critical role in stabilising the phase-locking between multiple solitons with remarkable quality, allowing formation of various types of bound-state solitons.

### Experimental results

Stable mode-locked pulse train self-starts when the PCs adjust the cavity birefringence and with >530 mW pump power. This value is higher than the threshold of a similar laser setup mode-locked with 1.25–1.5 nm diameter laser ablation SWNTs (350 mW)^34^ due to higher saturation intensity (I_*sat*_ ∼ 9.15 MW cm^−2^) of DWNTs, compared to that of the SWNTs (1.22 MW cm^−2^). After initiation, the mode-locked operation can be maintained over several hours under laboratory conditions with the insignificant fluctuation of output parameters. At >800 mW pump powers, we observe three different ultrashort pulse generation regimes by tuning the PC as predicted by numerical simulation.

#### Soliton generation

**Figure 6:**
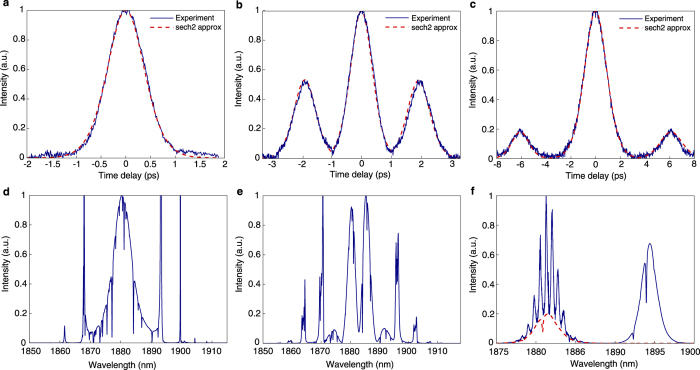
Laser output parameters showing (**a,d**) soliton, (**b,e**) soliton molecule, and (**c,f**) two-colour duplet + singlet regimes at 950 mW pump power; (**a–c**) measured autocorrelation traces; (**d–f**) output spectra. The measured autocorrelation traces and theoretical approximations with a sech^2^ shape we show in blue and dashed red, respectively. The dashed red plot in (**f**) shows spectrum baseline.

Spectrum and autocorrelation function of the conventional soliton generation at 950 mW pump power confirm single-pulse operation in Fig. 6(a,d). The autocorrelation trace in Fig. 6(a) is accurately fitted with ideal sech^2^ temporal profile (red dashed curve). The pulse full width at half-maximum (FWHM) is 560 fs. No secondary peaks are observed over the entire scan range (15 ps) of the autocorrelator. Figure 6b shows the output optical spectrum. We note that the Tm-doped fibre laser operation wavelength range (around 2 *μ*m) includes the water vapour absorption peaks. Since the OSA is not in the vacuum or nitrogen environment, the pulse spectrum is affected by environmental water vapour absorption during free-space light propagation. The output spectrum width at −3 dB is 6.3 nm. Sharp spectral side bands indicate stable soliton pulse shaping^76^ (compare with Fig. 5a). Together with the measured output spectrum, the time-bandwidth product of 0.314 justifies the generation of single transform-limited pulses. The average output power is 99.4 mW, corresponding to 1.66 nJ of total energy and 2.96 kW pulse peak power.

#### Bound state soliton generation

Multiple soliton operation is achieved at the same level of the pump power by adjusting the PCs. Once the bound-state soliton generation is obtained, the laser can operate stably for several hours in laboratory condition. The average output power is 63.15 mW, corresponding to 1.05 nJ of bound state soliton total energy. Figure 6(b,e) shows typical measured spectrum and autocorrelation trace of the obtained bound-state solitons. The autocorrelation trace demonstrates three peaks, with 1:2:1 intensity ratio. This is a clear indication of the existence of twin pulses with identical intensity and stable separation. The measured FWHM of each peak is 870 fs, giving 565 fs pulse duration, assuming soliton shape (Fig. 6b). The time separation between the bound soliton is ∼1.8 ps, which is 3 times larger than the pulse duration. Such a small time separation is an indication of the attractive or repulsive force exerted on the respective soliton due to direct soliton interaction, the sign of force being dependent on the phase difference between the two solitons^18^.

The optical spectrum of the bound solitons is presented in Fig. 6(e) (compare with Fig. 5b). The spectrum is modulated with high contrast (spectral intensity in minima is close to zero) due to the fixed coherent phase difference in the temporal domain between the two identical pulses. The spectral modulation interfringe is inversely proportional to the temporal separation of pulses 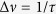. The period of the spectral modulation is 6.11 nm (Δ*ν* = 518.61 GHz). The calculated bound soliton separation based on Fourier analysis (1.92 ps) corresponds well to the experimental observation from the autocorrelation trace (1.8 ps). Comparing the spectra of fundamental soliton Fig. 6d and bound-state soliton in Fig. 6(e), we observe that the modulated optical spectrum of soliton molecule resembles the envelope of a single soliton with a red-shift of 2.5 nm in the central wavelength, preserving the position of the Kelly side-bands relatively to the central wavelength. Moreover, the phase shift between two pulses in the bound-state determines the symmetry of spectrum. The spectral modulation features a symmetric structure with a dip in the centre, showing that the phase difference between the bound solitons is +*π*, as theoretically predicted^77^. We note, that the peaks in Fig. 6e exhibit a minor asymmetry in the intensity (compare with Fig. 5b). Interestingly, the asymmetry is inverse for simulation and experimental results, i.e. the blue peak is more intense in the simulation, and the red one is more intense in the experiment. This is likely due to the small difference in group velocity of the bound-state solitons^26^ and therefore hopping between two states with the phase difference of π-and -π/2 in the experiment (π-and π/2 in the simulation)^25^. However, high contrast of spectral fringes and small asymmetry of both experimentally and numerically obtained spectra indicates longer lifetime of π-shifted bound solitons, comparing with ±*π*/2-shifted ones.

#### Two-colour soliton generation

By slightly adjusting the PC, we observe dual wavelength generation with a maximum total average power of 112 mW. (Fig. 6f). The central wavelengths of the spectral peaks are 1881.4 and 1894.3 nm. Thus, the separation between the two wavelengths is 12.9 nm. The peaks have a similar shape, with a 2.16 nm bandwidth but different intensities.

We note that the spectrum centred at 1881.4 nm consists of two contributions and represents the spectrum of a soliton pair and a soliton singlet^26^. As in previous cases the spectrum of the multi-soliton complex includes sharp spikes, attributed to the water vapour absorption. Such peaks are more indicative in the broad profile of the spectrum. The contribution of soliton pair spectrum demonstrates spectral modulation interfringe of 0.75 nm, in correspondence with a soliton pair separation of 6 ps. This value is in good agreement with the temporal separation, observed in the autocorrelation trace (Fig. 6e). The spectrum is modulated with the low contrast. The baseline of the spectral fringes, shown as dotted line in Fig. 6, corresponds to the spectrum of an additional single soliton pulse. The singlet is not synchronised with the two-soliton molecule and, therefore, does not introduce additional spectral modulation. The asymmetry of the spectral fringes implies that the soliton molecule and singlet soliton have different group velocities^26^. The autocorrelation trace features three peaks with 1.37-ps duration and 1:5:1 intensity ratio, which also indicate a complicated pulse dynamics in the cavity supporting interleaving of two different bound state structures^25^.

Although soliton pair + singlet generation is also observed numerically (Figs 4 and 5(d)), the existence of the second spectral peak at 1894.3 nm cannot be predicted with the current numerical model. It is worth mentioning that the baseline of the spectral fringes at 1881.4 nm matches the shape of the right peak of the output spectrum with high accuracy (red dashed line in Fig. 6(f)). We therefore argue that the right spectral peak corresponds to the soliton singlet. The origin of the stable dual-wavelength generation remains an open question and requires further investigation.

## Conclusion

In conclusion, we have experimentally evaluated properties of DWNT/PVA polymer composites as a saturable absorber. The unique double-wall structure gives DWNTs a larger modulation depth (64%) and faster carrier dynamics (with 1.25-ps relaxation time) than SWNTs, offering enhanced light-DWNT interaction. The present results suggest that DWNTs as SA are extremely effective for generating not only ultrashort conventional soliton pulses but also stable bound-state multi-soliton complexes beyond 1.8 *μ*m. This could be mainly attributed to their rigid structure, high modulation depth and faster carrier relaxation time, stabilising the phase-locking between multiple solitons.

Using hybrid mode-locking scheme with DWNTs and NPE, we have experimentally demonstrated the generation of conventional soliton pulses and bound states of solitons in the Tm-doped fibre laser. The laser configuration enables the formation of 560-fs fundamental soliton pulses with ∼100 mW average output power and 2.96 kW pulse peak power, exceeding the majority of previous experimental demonstrations of SWNT or DWNTs mode-locked Tm-doped fibre lasers. We have clear signatures of operation in a bound-state soliton regime. The observed soliton molecules are very stable, with the bound states having discrete, fixed soliton separation. The results of the numerical simulation are in good qualitative agreement with the experimental data and demonstrate the same regimes of laser generation: single soliton, bound-state soliton pair and singlet + doublet soliton structure. The numerical simulation showed that precise control of the net polarisation state together with pump power allows management of the number of pulses, their separation in the time domain and their phase shift in a wide range. The experiment demonstrated three numerically predicted regimes of conventional soliton and bound state soliton generation: two equal bound-state solitons with 560 fs and 1.37 ps pulse duration; and singlet with doublet soliton structures with duration of 1.8 ps and 6 ps pulse separation of the bound state in the time domain, respectively.

We anticipate that results we report here will pave the way for real-world applications, such as data carriers in optical communication allowing an increase in the bit-rate of data transfer, with the potential to exceed the Shannon limit.

## Additional Information

**How to cite this article:** Chernysheva, M. *et al*. Double-Wall Carbon Nanotube Hybrid Mode-Locker in Tm-doped Fibre Laser: A Novel Mechanism for Robust Bound-State Solitons Generation. *Sci. Rep.* **7**, 44314; doi: 10.1038/srep44314 (2017).

**Publisher's note:** Springer Nature remains neutral with regard to jurisdictional claims in published maps and institutional affiliations.
